# Exploring the prevalence of chlamydial and gonorrheal infections in pregnant women: a multicenter study in Egypt

**DOI:** 10.1186/s12889-024-20239-9

**Published:** 2024-10-16

**Authors:** Omaima El-Gibaly, Momdouh Wahba, Nahla Gamaleldin, Alaa Hashish, Mostafa Nasr Ibrahim, Ahmed Khedr Khalifa, Sherif Yehia Mohammed, Mohamed Ahmed wasfy, Sara Ali Mohammed Eldosky, Wagdy Amin, Heba Elsayed, Mariam Taher Amin

**Affiliations:** 1 Department of Public Health and Community Medicine, Faculty of Medicine, Assiut University, Assiut, Egypt; 2 Egyptian Family Health Society, Cairo, Egypt; 3 Department of Public Health and Community Medicine, Faculty of Medicine, Alexandria University, Alexandria, Egypt; 4 World Health Organization, Muscat, Oman; 5 Department of Obstetrics and Gynecology, Faculty of Medicine, Assiut University, Assiut, Egypt; 6 Department of Obstetrics and Gynecology, Faculty of Medicine, Beni-Suef University, Beni-Suef, Egypt; 7 Department of Clinical Pathology, Faculty of Medicine, Zagazig University, Zagazig, Egypt; 8 Department of Obstetrics and Gynecology, Faculty of Medicine, Zagazig University, Zagazig, Egypt; 9 Department of Obstetrics and Gynecology, Damitta Specialized Hospital, Damietta, Egypt; 10 National Tuberculosis control Program, Chest Diseases Department, Ministry of Health and Population, Cairo, Egypt; 11 National HIV&STIs Program, Ministry of Health and Population, Cairo, Egypt

**Keywords:** Sexually transmitted infections (STIs), Chlamydia trachomatis, Neisseria gonorrhoeae, GeneXpert CT/NG

## Abstract

**Background:**

Chlamydia trachomatis (CT) and Neisseria gonorrhoeae (NG) are widespread, treatable sexually transmitted infections (STIs) of global significance, affecting millions annually. Left untreated, they pose significant risks, including pelvic inflammatory disease (PID), infertility, and complications during pregnancy. The U.S. Centers for Disease Control recommends annual chlamydial screening for sexually active women to address these risks. Responding to this global challenge, the World Health Organization (WHO) has formulated a global health sector strategy on sexually transmitted infections, outlining priority actions to strengthen STI responses in countries. However, STI epidemiological studies encounter challenges in developing nations like Egypt due to socio-cultural factors, poverty, and limited diagnostic facilities. In Egypt, STI diagnosis primarily relies on clinical presentations, lacking structured screening programs for CT and NG. This study’s main objective is to estimate the prevalence of Chlamydial and gonorrheal infections, advocating for supportive STI strategies in Egypt. Additionally, the study aims to provide a foundation for national prevalence estimates of CT and NG infections.

**Methods:**

A cross-sectional study encompassed five antenatal clinics in different regions of Egypt. A total of 1040 pregnant women attending these clinics were consecutively sampled. Data collection involved structured questionnaires, and urine samples were subjected to the GeneXpert CT/NG qualitative real-time PCR test.

**Results:**

The prevalence of CT infections was 0.29% (95% CI, 0.10–0.86%), with no detected NG infections. The three CT-positive cases were distributed across different recruitment centers, with no statistically significant differences observed between infected and non-infected participants. Notably, 40.3% of recruited women reported gynecological symptoms, primarily discharge. Additionally, 9.6% had undergone previous testing for sexually transmitted infections, with 8.2% receiving positive results.

**Conclusions:**

This study provides valuable data on the prevalence of CT and NG infections among pregnant women attending ANC clinics in Egypt. The findings underscore the importance of ongoing surveillance, routine screening, and targeted interventions to ensure the reproductive health and well-being of pregnant women and their infants. Further research is warranted to explore the broader implications of STIs in different populations and to inform evidence-based guidelines for screening and management in diverse settings. **Trial registration**: IRB no.: 17,400,017; WHO ERC Protocol Id. A66005.

**Supplementary Information:**

The online version contains supplementary material available at 10.1186/s12889-024-20239-9.

## Background

*Chlamydia trachomatis* (CT) and *Neisseria gonorrhoeae* (NG) infections represent prevalent, curable sexually transmitted infections (STIs) globally [1, 2], infecting an estimated 129 million and 82 million people, respectively, in 2020 [3]. Both infections, often asymptomatic, can lead to serious reproductive health complications, including pelvic inflammatory disease (PID), which is a significant cause of infertility, ectopic pregnancy, and chronic pelvic pain in women [1]. As with other inflammatory STIs, CT and NG infections can facilitate the transmission of HIV infection [4]. Additionally, pregnant women with CT may transmit the infection to their infants during pregnancy, leading to premature birth and placental insufficiency. It may lead to ophthalmia neonatorum during delivery, leading to blindness and pneumonia [5]. Due to the significant health challenges and potential dangers linked with infection, the U.S. Centers for Disease Control (CDC) suggest annual screening for Chlamydia in sexually active women below the age of 25, as well as in women aged 25 and above who face an elevated risk of infection [1, 6].

To tackle this urgent worldwide challenge and empower nations to achieve the Sustainable Development Goals (SDGs), the World Health Organization (WHO) formulated the Global Health Sector Strategy on Sexually Transmitted Infections, spanning 2022 to 2030 (Global HIV, Hepatitis, and STIs Strategy). This comprehensive strategy outlines critical steps for countries to enhance their response to sexually transmitted infections (STIs), aiming to strengthen efforts, save numerous lives, and enhance the health of millions [7].

However, epidemiological studies of sexually transmitted infections (STIs) in developing countries face challenges, including socio-cultural factors (stigma), poverty, and inadequate diagnostic facilities [8]. In Egypt, the diagnosis of STIs predominantly relies on etiological and clinical presentations due to the absence of structured screening programs for CT and NG [9]. Complicating matters, both CT and NG can manifest asymptomatically, contributing to a significant underreporting of cases [4, 7].

Despite their asymptomatic nature, untreated CT and NG infections can lead to detrimental consequences such as damage to the female genital system and infertility [5, 10]. The persistence of CT averages around 1.4 years, while NG can linger for about six months without antimicrobial treatment. Notably, the longer the clearance time, the higher the prevalence of untreated infections, necessitating effective screening interventions [4, 8].

The Egyptian Family Health Survey 2021, a national survey focusing on women in their reproductive years, sheds light on the prevalence of STIs. Of the surveyed women, only 1% reported that they had acquired STIs in the past 12 months. Additionally, one-fourth of women reported abnormal genital discharge [11].

Challenges in screening for CT and NG lie in the fact that the gold standard for diagnosis in a culture is a method demanding genital swabs. Recent advancements in diagnostic techniques, such as the Xpert CT/NG assay on the GeneXpert system, offer a more accessible approach [12, 13]. This nucleic acid-based test aligns with WHO REASSURED criteria for STI diagnostic tests, offering rapid results within 90 min, real-time connectivity, ease of specimen collection, affordability, sensitivity, specificity, user-friendliness, rapidity, robustness, equipment simplicity, environmental friendliness, and deliverability to end-users [12, 14].

Previous studies in Egypt mainly targeted symptomatic individuals or those already diagnosed with STIs, aiming to understand the etiology of infections. Similarly, studies among infertile women aimed to discern the role of infections in infertility. Due to difficulties in reaching sexually active women for prevalence studies, pregnant women are often considered proxies [8].

The absence of comprehensive epidemiological data on these infections poses a significant challenge in evaluating the disease burden, impeding the development of effective prevention and therapy programs, proper resource allocation, and targeted advocacy plans. This gap becomes particularly noticeable when contrasted with the prioritization and exclusive resource allocation for HIV infections. It underscores the potential cost-effectiveness of integrating other STIs into comprehensive intervention strategies. Therefore, this study seeks to determine the prevalence of CT and NG, specifically among pregnant women in Egypt. This research aims to contribute valuable insights into the broader epidemiology of STIs in the country.

## Methods

### Study design and setting

This multicenter cross-sectional study was conducted at five antenatal clinics representing diverse regions across Egypt. The investigation aimed to assess the prevalence of Chlamydia trachomatis (CT) and Neisseria gonorrhoeae (NG) infections among pregnant women attending antenatal care (ANC) clinics.

### Study population

The study included pregnant women attending ANC clinics at the following centers: Kafr Nasar Maternal and Child Health Centre in Giza (Greater Cairo), Mansoura University Hospital (Delta region), Assiut University Hospital (Upper Egypt), Zagazig University Hospital (Lower Egypt), and Beni-Suef University Hospital (Upper Egypt).

### Inclusion criteria

The inclusion criteria included pregnant women attending the clinic for the first time during their current pregnancy, consenting to participate, and having the ability to provide urine samples for testing.

### Exclusion criteria

Exclusion criteria were pregnant women who had previously visited the clinic during the survey period, those not providing informed consent, and women who had used antibiotics, douches, or vaginal creams within the last 24 h.

### Sample size

To ensure a representative sample, the sample size was calculated using Epi-Info software version 3.5.1 [15]. , incorporating assumptions of an expected frequency of 10% [8], a margin of error of 2%, and a confidence interval of 95%. The initially estimated sample size was 864 pregnant women, with a 20% increase applied, resulting in a final sample size of 1040.

### Study instruments

A structured questionnaire was developed to collect participant data and laboratory tests for urine samples.

### Structured questionnaire

The questionnaire was specifically developed for this study following a comprehensive review of existing literature to effectively gather data from participants through interviews conducted by research investigators. It covered socio-demographic and reproductive data (residence, age, education, work status, parity, etc.), the presence or absence of symptoms of infection (discharge, pain during intercourse, etc.), previous STIs, and extrauterine pregnancy. The questionnaire is provided in supplementary file 1.

### Urine sample collection and testing

Urine samples were collected following specific guidelines for GeneXpert kit analysis [16]. Each participant received a unique identifier number, written on a sterile cup provided by the lab to ensure traceability. Participants were instructed to avoid cleaning the genital area and to catch the first-void urine in the sterile cup. An appropriate transport medium (Xpert CT/NG urine transport reagent tube) was used to stabilize nucleic acid until examination. The samples were preserved in the refrigerator at the study site before weekly transportation to the MoHP TB laboratory. The urine samples underwent Xpert CT/NG qualitative real-time PCR test for the automated detection and differentiation of CT and NG, run through a GenExpert Platform (Kits provided by Cepheid Co.). Results were typically obtained after 90 min.

### Pilot study

A pilot study was conducted to validate the questionnaire and refine data collection procedures, including the logistics of urine sample collection, transportation, and laboratory analysis.

### Data collection

Data were collected from August 2022 to September 2023. At each study site, a research investigator (a physician providing care at the clinic), assisted by the clinic nurse, conducted interviews and collected urine samples. The samples were labeled, and the investigators followed the instructions for preserving and transporting them to the laboratory. All investigators received training on the questionnaire and procedures for collecting urine samples, transporting them to the lab, and collecting the results of the urine examination, linking them to each participant’s questionnaire.

### Data management and analysis

Data management involved Excel and RStudio software. After immediate review for completeness and accuracy, forms were sent to Assiut University for data entry. Double data entry, validation, and cleaning were performed before statistical analysis.

### Statistical analysis

Descriptive statistics were used to characterize the sample, including frequencies, percentages, mean ± standard deviation, and median (minimum-maximum).

The prevalence of infection was calculated, and the 95% confidence interval was determined using the Wilson score interval method, which is particularly suitable for low prevalence [17].

### Ethical considerations

The study protocol was reviewed and approved by the Institutional Review Board of the Faculty of Medicine, Assiut University (IRB no.: 17400017) and the WHO Research Ethics Review Committee (WHO ERC) (Protocol Id. A66005).

#### Informed consent

was obtained from all participants prior to their involvement in the study. In cases where participants were illiterate, informed consent was obtained from appropriate representatives. The study adhered strictly to ethical guidelines, ensuring participant privacy and confidentiality throughout the research process.

Furthermore, individuals diagnosed with infections received free treatment following WHO guidelines, underscoring our commitment to ethical and equitable care for all participants.

## Results

**Fig. 1:**
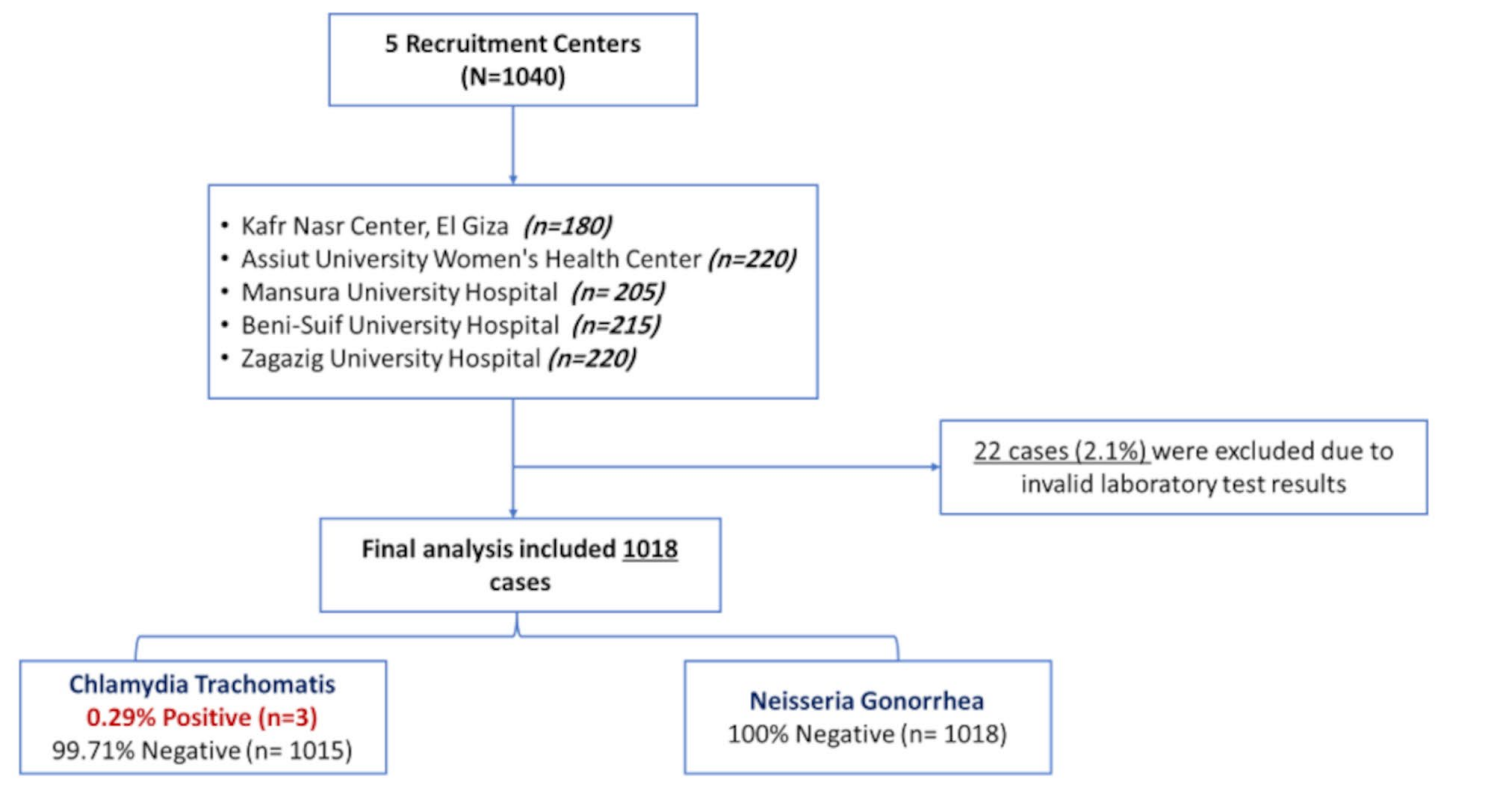
Flow diagram of the study

The study enrolled a diverse cohort of pregnant women across five antenatal care centers in Egypt: Assiut (21.2%), El-Giza (17.3%), Mansoura (19.7%), Beni-Suef (20.6%), and Zagazig (21.2%). However, 2.1% of the specimens were initially reported either as “error,” “invalid,” or “no result” by the Xpert test, so the results could not be reported as either positive or negative and have been excluded from the analysis. The flow of participants through the study is presented in Fig. 1.

The socio-demographic characteristics of the recruited women revealed a mean age of 27 years, with nearly 58.5% falling within the 20–29 age group. Most participants were married before age 30 (97.4%), and approximately one-third resided in urban areas (32.7%). Educational attainment varied, with 44.6% completing secondary school or vocational education. Housewives constituted the predominant occupation among participants (90%). Further details on socio-demographic characteristics are presented in Table 1.

**Table 1:** Socio-demographic characteristics of recruited women

Characteristics	Frequency	%
Age (years)		
Mean ± SD	27.2 ± 6.1	
< 20	82	8.1
20–29	595	58.6
30–39	311	30.6
≥ 40	28	2.8
Age at marriage (years)		
Mean ± SD	19.7 ± 3.8	
≤ 18	466	45.8
19–29	525	51.6
≥ 30	26	2.6
Residency		
Urban	330	32.7
Rural	678	67.3
Education		
Illiterate/read and write	177	17.4
Completed primary/preparatory school	249	24.5
Completed secondary/Vocational education	453	44.6
University graduate or higher	137	13.5
Husband’s Education		
Illiterate/read and write	215	21.2
Completed primary/preparatory school	150	14.8
Completed secondary/Vocational education	481	47.4
University graduate or higher	165	16.3
Do not Know	4	0.39
Work		
Housewife	920	90.4
Paid work	91	8.9
Other	7	0.69
Husband’s Work		
Not Working	15	1.5
Employee	228	22.4
Skilled/Unskilled worker	563	55.4
Private business	207	20.4
Others	4	0.39

Reproductive characteristics of the participants revealed a mean pregnancy duration of 27 weeks, with the majority in the third trimester (56.1%). The median gravidity was 3, with 19.1% being primigravida. Most women reported no history of ectopic pregnancy (97.8%).

**Fig. 2:**
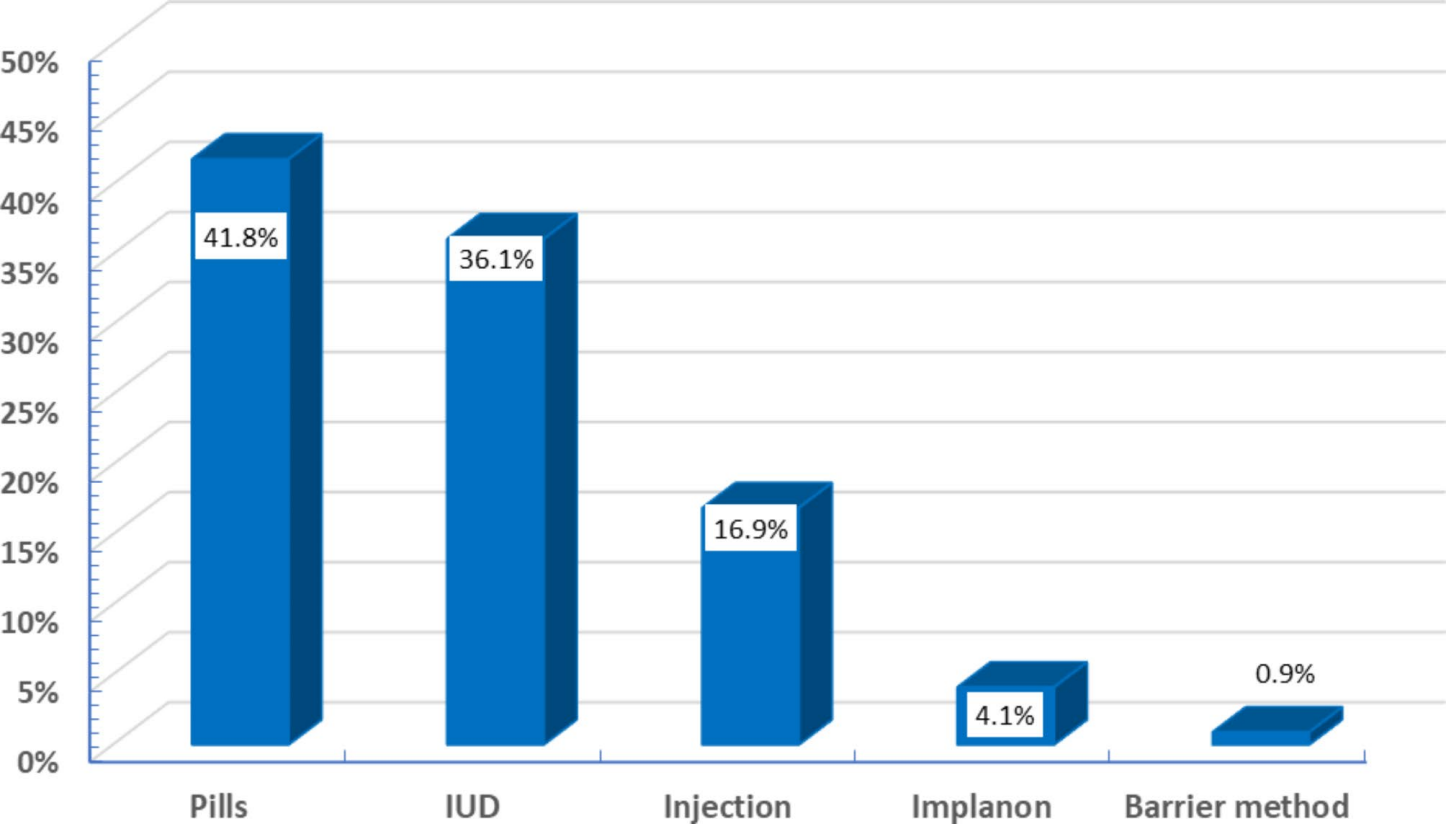
Distribution of last used contraceptive method

Concerning contraception practices, more than half of the participants (58%) reported using contraception before their current pregnancy. Pills and intrauterine devices (IUDs) were the prevalent choices, constituting 41.8% and 36.1%, respectively (Fig. 2).

**Fig. 3:**
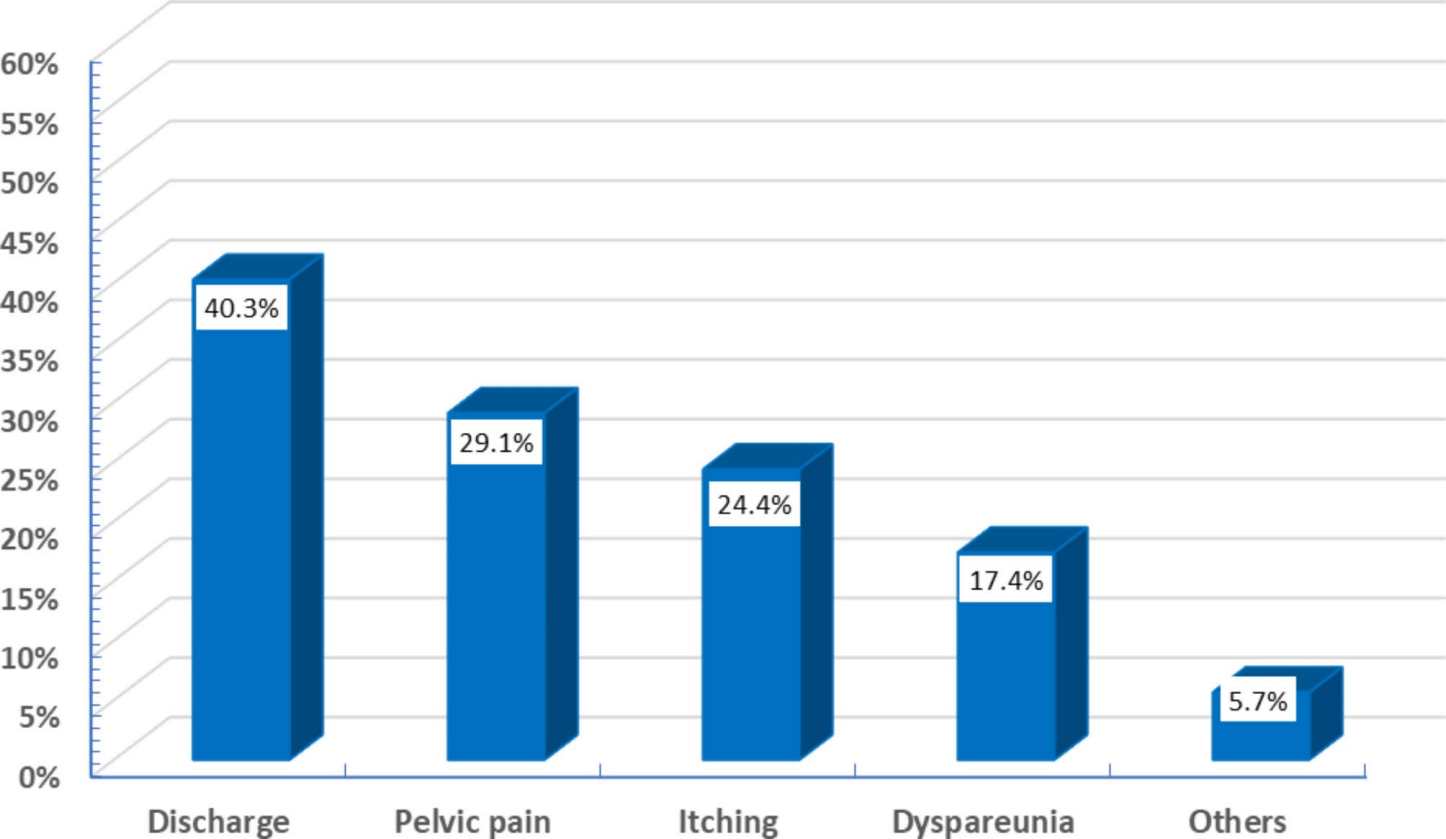
Current presentation of gynecological symptoms

Participants exhibited various gynecological symptoms, with discharge being the most reported (40.3%). Other prevalent symptoms included pelvic pain (29.1%), itching (24.4%), and dyspareunia (17.4%) (Fig. 3).

Previous testing for sexually transmitted infections (STIs) was reported by 9.6% of participants, with 8.2% receiving positive results, though the specific infection was not remembered. Among husbands who underwent STI testing, 3.1% had positive results. Notably, 76.8% of participants did not recall undergoing previous STI testing (Table 2).

**Table 2:** Previous STIs test for women or her husband

Characteristics	Frequency	%
Previous STIs test		
Yes	98	9.6
No	782	76.8
Do not remember	138	13.6
Result of previous STIs test		
Positive	8	8.2
Negative	72	73.5
Do not know	18	18.4
Husband’s previous STIs test results		
Positive	3	3.1
Negative	42	43.8
Do not know	51	53.1

**Fig. 4:**
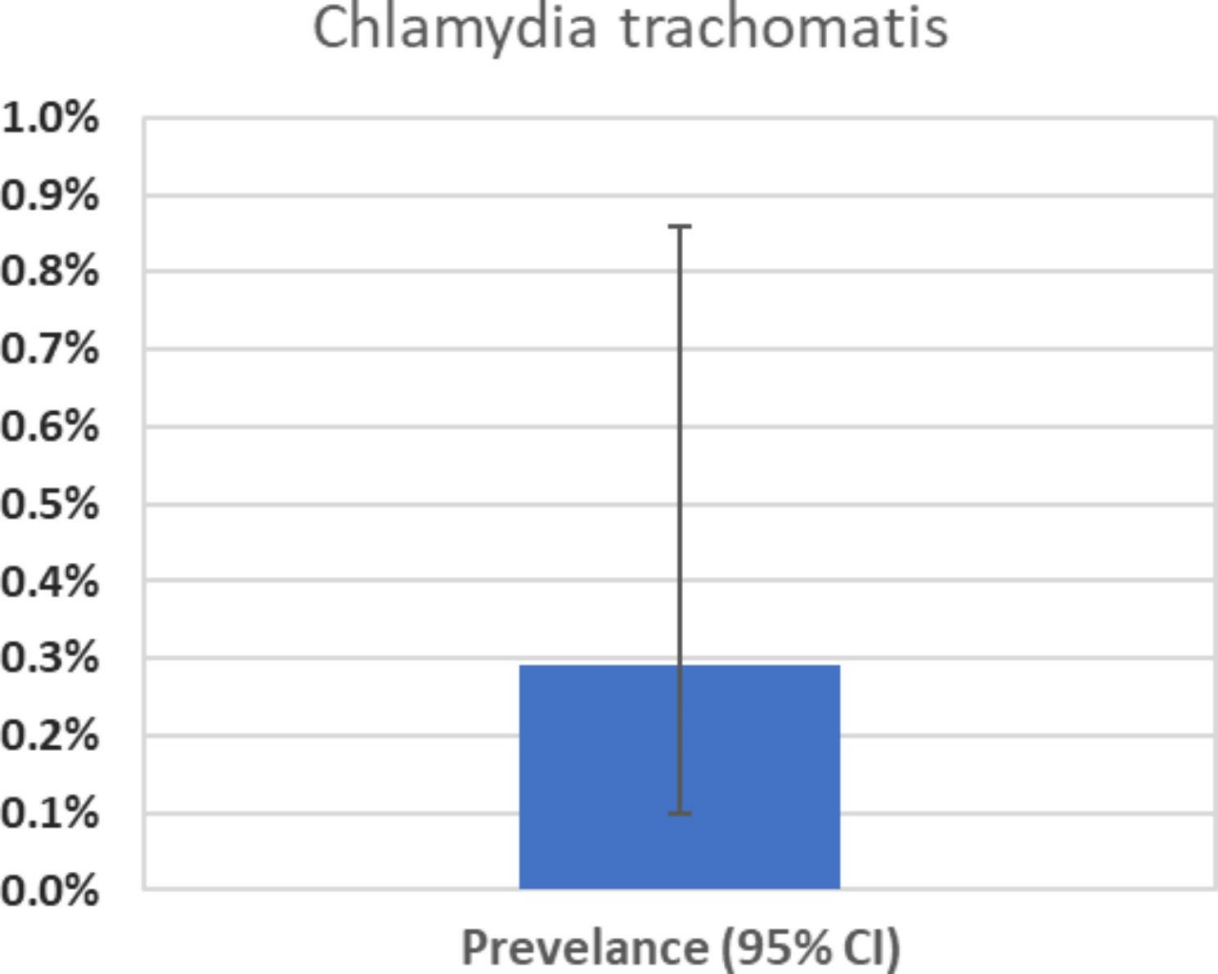
CT prevalence with 95% CI

The prevalence of CT infections among the study participants was 0.29%, with confidence intervals ranging from 0.10 to 0.86% (Fig. 4). Notably, no NG infections were detected in the study.

The three positive cases for CT were distributed across different recruitment centers, with one each in El-Giza, Mansoura, and Beni-Suef. In the third trimester of pregnancy, these cases were 29, 18, and 31 years old. Due to the low number of cases, no statistically significant differences were observed when comparing participants with STIs to the non-infected group.

## Discussion

The present study aimed to investigate the prevalence of Chlamydia trachomatis (CT) and Neisseria gonorrhoeae (NG) infections among pregnant women attending antenatal care (ANC) clinics in various regions of Egypt. The findings offer essential insights into the epidemiology of these sexually transmitted infections (STIs) in a population vulnerable due to the potential consequences of maternal infections on both maternal and neonatal health.

To our knowledge, the current study is the first investigation in Egypt specifically focused on determining the prevalence of CT and NG among pregnant women, serving as a proxy for sexually active women. Previous research in the country predominantly targeted individuals with STI symptoms or those already diagnosed, with a focus on specific types of infections or cases related to infertility [9, 10, 18, 19]. The overall prevalence of CT infections among the study participants was 0.29%, and no cases of NG were detected.

In contrast, the 2021 Egyptian Family Health Survey inquired about STI history and genital discharge in the past year among women. The results indicated that only 1% of women reported having a sexually transmitted infection in the 12 months preceding the survey [11]. Regarding STI symptoms, 25% reported abnormal genital discharge, while the present study found that 40% reported having a discharge.

A study at Mansoura University Hospital aimed to detect C. trachomatis in Egyptian women with genital infection symptoms, revealing a positive CT cell culture in 22.8% of symptomatic cases [20]. Additionally, a study on Chlamydia trachomatis screening among Egyptian women with unexplained infertility showed a 6% prevalence of current Chlamydial infection [18]. Another investigation among infertile women at Suez Canal University Hospital found a 2.7% prevalence of CT and 2% of NG [10].

In 2020, a study in Assiut explored the prevalence of various STIs in married women, identifying common infections such as candidiasis, scabies, genital warts, and trichomoniasis. Neisseria gonorrhoeae was present in 5% of cases, though Chlamydia trachomatis was not investigated [9].

Despite variations in prevalence across studies, the low prevalence of CT and NG in Egypt aligns with rates in other Middle East and North Africa (MENA) region countries. A meta-analysis reported pooled prevalence rates: 3.0% in general populations such as antenatal clinic attendees, blood donors, and pregnant women; 2.8% in intermediate-risk populations, which include individuals who are presumed to have some sexual contact with groups engaging in high-risk sexual behavior; 13.2% in female sex workers; 11.3% in infertility clinic attendees; 12.4% in women with miscarriage; and 12.4% in symptomatic women [8].

Several factors may explain this. Firstly, the region lacks comprehensive screening for these infections, leading to diagnosed cases representing only the tip of the iceberg. In particular, silent infections, such as CT, may persist without symptoms for extended periods, contributing to underreporting [2, 6, 8].

A predominantly symptomatic diagnosis approach further influences the low prevalence. In cases where individuals complain of pelvic pain or discharge, antibiotics are often prescribed without thorough consideration of the specific infection causing the symptoms [4, 8]. Compounding this issue, the accessibility of antibiotics as over-the-counter drugs in our community contributes to their irrational use [2].

Additionally, seeking medical advice for sexually transmitted infections (STIs) may carry a stigma in our culture. Consequently, these infections are likely underreported in the Middle East due to a reluctance to disclose such information [8, 21].

The presence of symptoms like discharge, pelvic pain, and itching emphasizes the urgent need for comprehensive reproductive health services and education, particularly for pregnant women. Addressing these challenges requires a multifaceted approach, encompassing improved screening, judicious antibiotic use, and destigmatizing conversations surrounding STIs in the Middle East.

This study significantly contributes to the field by employing urine analysis to detect chlamydial and gonorrheal infections among pregnant women. The decision to utilize urine samples, as opposed to traditional genital swabs, is notable and provides practical advantages that enhance the feasibility and acceptability of screening in diverse healthcare settings. The non-invasive nature of urine sample collection streamlines the screening process and increases participation rates, making it an accessible and patient-friendly method. This approach aligns with recent advancements in non-invasive testing methods for sexually transmitted infections (STIs) and marks a positive step toward overcoming barriers to screening [12, 22].

The validity of the GeneXpert platform for STI detection has been established in prior studies. The qualitative real-time PCR test conducted through the GeneXpert system ensures automated and accurate detection and differentiation of CT and NG [13, 14]. The GeneXpert system is an automated molecular diagnostic method based on nucleic acid amplification testing developed by Cepheid in the USA. Its detection technology relies on a 90-minute real-time fluorescent qualitative polymerase chain reaction (FQ-PCR), exhibiting comparable performance to traditional nucleic acid amplification tests [12, 23]. The Xpert platform seamlessly integrates and automates various processes, including sample preparation, nucleic acid purification, gene amplification, and result reporting through qualitative PCR molecular detection. This enables the rapid detection of CT and NG pathogens on the same day [14].

The Xpert system offers numerous advantages, including the independent use of each machine module and the simultaneous screening of multiple samples. It is faster, simpler, accurate, and safer than traditional methods. A meta-analysis substantiates the high diagnostic accuracy of Xpert CT/NG for both CT and NG. Notably, the system demonstrated elevated sensitivity and specificity in detecting anorectal, urethral, and vaginal samples without significant differences based on gender. Consequently, Xpert CT/NG can potentially emerge as the primary method for detecting CT and NG, possibly evolving into the gold standard for diagnosis in the future [13].

The strengths of this multicenter study lie in its representative sample, which included various regions of Egypt. Significantly, the study enhances our understanding of chlamydial and gonorrheal infections among pregnant women as a proxy for sexually active women, emphasizing the necessity for a nuanced approach to STI research and prevention.

However, it is essential to acknowledge potential confounding factors, such as the widespread use of antibiotics, particularly azithromycin, during the peak of the COVID-19 epidemic. This prevalent usage, recommended by healthcare providers and available over-the-counter, may impact the observed low prevalence rates of the studied STIs, given their known sensitivity to this antibiotic.

Furthermore, the absence of comprehensive STI surveillance in Egypt underscores the imperative for an enhanced national system to monitor and effectively address STIs. This highlights the diversity of STIs in different populations and underscores the importance of tailored screening and management approaches. Future research should aim for a comprehensive understanding of STIs in diverse populations, informing targeted preventive measures and interventions.

## Conclusions

In conclusion, this study contributes valuable data on the prevalence of CT and NG infections among pregnant women attending ANC clinics in Egypt. The findings underscore the importance of ongoing surveillance, routine screening, and targeted interventions to ensure the reproductive health and well-being of pregnant women and their infants. Further research is warranted to explore the broader implications of STIs in different populations and to inform evidence-based guidelines for screening and management in diverse settings.

## Electronic supplementary material

Below is the link to the electronic supplementary material.

Supplementary Material 1

## Data Availability

The questionnaire and the datasets used and/or analyzed during the current study are available from the corresponding author upon reasonable request.

## Declarations

Furthermore, individuals diagnosed with infections received free treatment following WHO guidelines, underscoring our commitment to ethical and equitable care for all participants.
