# Supplementary material for: Exploring the prevalence of chlamydial and gonorrheal infections in pregnant women: a multicenter study in Egypt
Source: BMC Public Health. 2024 Oct 16;24:2852. doi: 10.1186/s12889-024-20239-9 (PMC11484395; doi:10.1186/s12889-024-20239-9)
Supplement: Supplementary file 1 — Supplementary Material 1 [file 12889_2024_20239_MOESM1_ESM.pdf]

Supplementary file 1. Study tools

A. Clinic data collection form (The questionnaire)

1. Hospital code: [ ]
    1. Kafr Nasar Centre- Faisal Street, Giza
    2. Mansoura University
    3. Zagazig University
    4. Assiut Universit
    5. Bani-Suef University

2. Participant ID number: [ ][ ][ ][ ]

3. ANC record number: [ ][ ][ ][ ]

4. Way for contacting to give results:…………………………….Phone no.:…………………

5. Date of specimen collection (day/month/year): _______/______/________

6. Age or Date of birth (day/month/year): ______/______/________ (_____ age in years)

7. Age of marriage (_____ age in years)

8. Duration of current gestation: _______________ weeks

9. Total number of pregnancies: _______________

10. Total number of live births (babies born alive): _____________

11. Number of pregnancies that did not end in a live birth (spontaneous abortion, elective abortion, or stillbirth): _____________

12. History of previous ectopic pregnancy:
    1. Yes (------ times)
    2. No
    3. Don't remember/ know

13. Previous contraceptive use:
    1. Yes
    2. No

If Yes (specify):…………………..

1. IUD
2. Hormonal pills (COCs , POPs)
3. Implanon
4. Injectable counteaception
5. barrier methods, such as condoms.
6. Other

14. Place of residence:
    1. Urban
    2. Rural

15. Education level of woman
    1. No education
    2. Some primary education
    3. Completed primary education
    4. Some college or university
    5. Completed university

16. Education level of husband
    1. No education
    2. Some primary education
    3. Completed primary education
    4. Some college or university
    5. Completed university

17. Employment status of woman
    1. Salaried
    2. Self-employed
    3. Unemployed
    4. No answer
    5. Others (……………….)

18. Employment status of husband
    1. Salaried
    2. Self-employed
    3. Unemployed
    4. No answer
    5. Others (……………….)

19. Have you ever tested for STIs?
    1. Yes
    2. No

20. Husband's STI status (self-reported).

1. Positive
2. Negative

If positive (specify):…………………..

21. Current complaint of the women (if any)………………………………..

1. Discharge
2. Pruritis
3. Intercourse pain
4. Pelvic pain
5. Others (……………………)

22. Vaginal hygiene practices

1. No specific practice
2. Regular cleaning with plain water
3. Vaginal douche
4. Others (………………………..)

B. Laboratory data collection form

Laboratory name: ______________________________

Name of test: _________________________________

Manufacturer: ________________________________

Lot number: __________________________________

Expiry date (day/month/year): ______/_____/__________

Results read by: ☐ Staff Name 1 ________ ☐ Staff Name 2 _________

Participant number	Hospital Name	Date of specimen received (day/month/year)	Date when test done (day/month/year)	Lab Results	
				Chlamydia	Gonorrhea
		/ /	/ /		
		/ /	/ /		
		/ /	/ /		
		/ /	/ /		
		/ /	/ /		
		/ /	/ /		
		/ /	/ /		
		/ /	/ /		
		/ /	/ /		
		/ /	/ /		
		/ /	/ /		

P = positive; N = negative; I = invalid (The test with invalid result should be repeated with a new test and the result will be recorded as "invalid" if the result of a repeated test is still invalid).
